# Novel +90G>A Intronic Polymorphism of *CYP2D6*

**DOI:** 10.22074/cellj.2015.514

**Published:** 2015-04-08

**Authors:** Monir Modaresi-nejad, Marzieh Shiva, Parvaneh Afsharian

**Affiliations:** 1 Department of Biology, Science and Research Branch, Islamic Azad University, Tehran, Iran; 2 Department of Endocorinology and Female Infertility at Reproductive Biomedicine Research Center, Royan Institute for Reproductive Biomedicine, ACECR, Tehran, Iran; 3 Department of Genetics at Reproductive Biomedicine Research Center, Royan Institute for Reproductive Biomedicine, ACECR, Tehran, Iran

**Keywords:** *CYP2D6*, Polymorphism, Novel Variant

## Abstract

**Objective:**

CYP2D6, an enzyme, metabolizes a large number of commonly prescribed drugs. Variations in *CYP2D6* gene encoding this enzyme have been associated with individual differences in drug metabolism rates. The purpose of our study was to identify some allelic variants of *CYP2D6* gene and to detect defective *CYP2D6* alleles, as part of a pharmacogenetic screening program.

**Materials and Methods:**

A prospective study was done on 120 participants referred to Royan Institute in 2013. Allele and genotype frequencies for polymorphism of *CYP2D6* gene in exons 1 and 4 were determined by polymerase chain reaction-restriction fragment length polymorphism (PCR-RFLP) analysis and sequencing on PCR products, respectively.

**Results:**

We identified a novel variant of the gene encoding cytochrome P450 2D6 (*CYP2D6*) at position +90 of intron 4 by sequencing method. This novel polymorphism of *CYP2D6* has been deposited in GeneBank^®^ under the accession number KF225465 in Jun 2013.

**Conclusion:**

In the current study, we identified novel polymorphism in intron 4. This single nucleotide polymorphism (SNP) is known as +90G>A in the fourth intron.

## Introduction

Cytochrome P450 (CYP), containing 57 functional genes and 58 pseudogenes, is primarily expressed in hepatic tissue. Among different CYPs in hepatic tissue, CYP2D6 accounts for approximately 2% of total CYP450 enzymes. However, it has been reported that 20-30% of prescribed drugs are metabolized by CYP2D6, including antidepressants, antiarrhythmics, antihypertensives and antipsychotics (1).

The *CYP2D6* gene cluster, which is located on chromosome 22q13.1 and lies adjacent to the *CYP2D7* and *CYP2D8* pseudogenes, has been found to have more than 300 genetic variants and 128 different alleles (2).

CYP2D6*10 is predominant in Asian populations, ranging from 30 to 50%, while CYP2D6*4 distinguishes Caucasians from other populations with a frequency of 12-21% (3). The most common deficient allele in Asians (allele frequency of >50%) and perhaps the most common *CYP2D6* allele in the world is CYP2D6*10 after CYP2D6*1 (4). The CYP2D6*10 allele, which results in Pro34Ser (P34S) substitution (C100T) in exon 1 and produces a low-activity enzyme (poor metabolizer; PM), has a frequency as high as 45 and 38.1% in the Korean and Japanese populations, respectively, whereas it has a low frequency (1.5%) in Caucasians (5, 6). The frequencies for CYP2D6 alleles *2 and *10 are 32 and 9%, respectively, within Eastern Azerbaijan (EA) population, a province in Northwest of Iran. Although allele frequency of CYP2D6*2 in Iranians (EA;-32%) has been reported almost similar to its frequency in Caucasians, their results in *10 allele frequency (EA; 9%) are more close to South Indians (10.2%) and Central Italians populations (8.1%) (7).

The most frequent inactivating mutation among Caucasians is the splice-site mutation G1846A defining the CYP2D6*4 allele (former type B allele) between exons 3 and 4, resulting in loss of enzyme activity (8). The frequency of this poor metabolizer phenotype in Caucasians has been reported 7-10% as a consequence of CYP2D6*4 (1846G>A) (9). The allele frequency for CYP2D6*4 has been reported 9% for Mazandaran population, a province in North of Iran (10), and 12.5% for EA population (7).

The PM phenotype can be due to two nonfunctional (null) alleles, whereas the extensive metabolizer phenotype is usually due to one or two alleles with normal function, such as CYP2D6*1 and CYP2D6*2 (11).

The data reported in present study were as part of a pharmacogenetic screening program in order to identify functional and non-functional *CYP2D6* alleles, such as CYP2D6*10, in 120 Iranian individuals. Furthermore we detected the alterations in amino acids of active site, e.g. Glu216 (in exon 4), in *CYP2D6*. During our screening, we identified a novel polymorphism (+90G˃A) in intron 4 of *CYP2D6*.

## Materials and Methods

This prospective study was conducted in Royan Institute, during 2013. DNA samples from 120 Iranian individuals with different ethnicities were examined. In this study, the ethnicity was defined as ethnicity of both participant and his/her parents. The Ethics Committee of Royan Institute approved the study. Cases signed an informed consent.

### Polymerase chain reaction-restriction fragment length polymorphism (PCR-RFLP) analysis

Genomic DNA was isolated from peripheral blood using the salting out method (12). Identification of CYP2D6*10 was performed based on RFLP on PCR products of exons 1. To evaluate the exon 4, sequencing was used on a fragment that was amplified by PCR from intron 3 to intron 4.

The PCR primers were designed by primer 3 program (http://primer3.ut.ee/) to amplify exons 1and 4 of *CYP2D6* gene and their neighboring introns in order to detect specific polymorphisms in *CYP2D6* gene. Primers were purchased from Pishgam Co., Iran. The sequences of primers have shown in table 1.

The PCR reactions were performed in a total volume of 50 μl containing 30 ng genomic DNA (final concentration of 0.6 ng/μl in each reaction), 1-1.5 mM MgCl_2_ (Cinagene, Iran), 0.2 mM dNTP-s (Cinagene, Iran), 0.4 pM of each primer (Sigma, USA) and 0.06 U/μL Taq DNA polymerase (Cinagene, Iran).

PCR protocol was consisted of 3 steps as follows: primary denaturation at 94˚C for 4 minutes continued by 30 cycles at 94˚C for 30 seconds, annealing at 62˚C for 30 seconds, extension at 72˚C for 1 minute, and final extension at 72˚C for 10 minutes by a thermo-cycler (Eppendorf, Germany). After amplification, the PCR products were assessed by 1.2% agarose gel electrophoresis (Paya Pazhoh Pars Co., Iran) and DNA was visualized by ethidium bromide (Sigma, USA) staining and Molecular Imager^®^ Gel Doc™ XR+ (BioRad, USA).

### RFLP patterns

The PCR products of exon1 were digested by *HphI* (Fermantas, Germany) at 37˚C for at least 6 hours to detect CYP2D6*10 (P34S) (C/T, rs1065852). RFLP patterns showed 363, 71, and 69 bp fragments for C allele as well as 263, 100, 71, and 69 bp fragments for T allele. The size of the digestion products was evaluated by 1.52% agarose gel electrophoresis that was followed by ethidium bromide staining.

Some samples of exon 1 were selected randomly to be sequenced in order to confirm the genotypes obtained by PCRRFLP analysis.

Purified PCR products of exon 4, including part of intron 3 and intron 4, were sequenced by an applied biosystem automated DNA sequencing Sanger method (Macrogen Inc., Korea). Also some samples of exon 1 were randomly sequenced. Finch TV software version 1.4.0 was used to analyze the sequencing diagram results (http://www.geospiza.com/Products/finchtv.shtml). In addition, results of each exon or intron were blasted against the ancestral sequence in NCBI (http://blast.ncbi.nlm.nih.gov.com).

## Results

**Fig.1:**
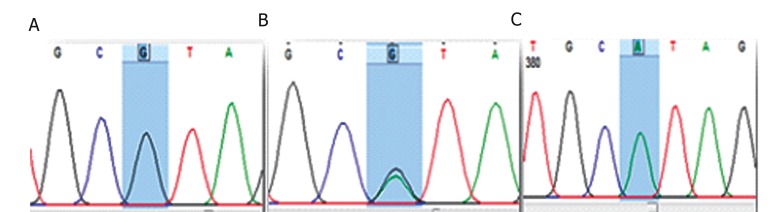
Chromatograms from Finch TV DNA sequencing of the CYP2D6, novel polymorphism in intron 4. A. Wild type, B. Heterozygote and C. SNP. The ID of SNP in nucleotide NCBI is KF225465. SNP; Single nucleotide polymorphism, ID; Identification and NCBI; National Center for Biotechnology Information.

On the basis of the results from the sequencing analysis, 95 individuals (79.17%) were homozygous (AA) at position +90 of intron 4 and they also showed to have ancestral sequence. Twenty five individuals (20.83%) had G allele in two different type of genotypes (Table 2) including heterozygous genotype (GA) that was detected in 13 individuals (11%), while the homozygous one (GG) was present in 12 individuals (10%) who showed alteration in both alleles (Fig.1). Our results were confirmed by double sequencing with both forward and reveres primers. In general, frequency of G allele as a novel single nucleotide polymorphism (SNP) in our population was 15.4%. This novel polymorphism of CYP2D6 has been deposited in GeneBank^®^ under the accession number KF225465 in Jun 2013.

Our data from RFLP method showed 10.8% of our population revealed to have P34S (Prolin34Serin) (rs1065852) polymorphism (CYP2D6*10) and 13.75% had T allele SNP (data not shown).

**Table 1:** Primer sequences of exons 1, 4 (including part of intron 3, exon 4 and intron 4) in CYP2D6 gene


Exon	Forward primer	Reverse primer

**1**	5´-GTCAACACAGCAGGTTCACTCAC-3´	5´-GTATAAATGCCCTTCTCCAGGAAGT-3´
**4**	5´-AAGAAGTCGCTGGAGCAGTG-3´	5´-AATCTCTGACGTGGATAGGAGGT-3´


**Table 2:** Comparison of genotype and allele frequency of different ethnicities in Iranian participants


		Genotype N(%)		Allele frequency (%)
Ethnicity	N (%)	GA	AA	A

**Fars**	60(50)	10 (16.7)	7(11.7)	20
**Azari**	22(18.3)	-	3 (13.6)	13.6
**Kord**	10(8.3)	1(10)	1(10)	15
**Gilak**	5 (4.2)	1(20)	-	10
**Lor**	8 (6.7)	1(12.5)	1(12.5)	18.8
**Arab**	5 (4.3)	0	0	0
**Others**	5 (4.3)	0	0	0
**Total**	120 (100)	13(11)	12 (10)	15.4


N; Number.

## Discussion

Inter-individual variability in pharmacokinetic may describethe significant variety in drug responses observed in medical setting. Response may be observed both in terms of distinct adverse drug reactions (ADRs) and inability to reach beneficial levels (13).

In a study conducted about pharmacokinetic variability, existent of genetic mutations in drug-metabolizing enzymes have been the predominant emphasis of pharmacogenetic studies. Due to the complication and enormous number of mutations present in these genes, the Human CYP Allele Nomenclature website was generated in order to register genetic variability in CYP enzymes (www.cypalleles.ki.se/) (14).

With the rapid development in sequencing technologies, DNA arrangement finding is improving which will allow the new techniques to form the basis of pharmacogenetics in the future. This will simplify simultaneous identification of different alleles in populations (15).

A SNP is a single nucleotide change in the DNA sequence and is observed in more than 1% of the population.

Our results suggest a novel polymorphism of *CYP2D6* since it was observed in 15.4% of our population, although it should be studied in different pure races of Iranian population and in functional point of view, as well. The occurrence of the novel polymorphism of *CYP2D6* may be limited to one population, or it may be present in more populations at low frequencies. The occurrence of *CYP2D6* polymorphisms, which are undetected until now, may explain sporadic existences of unexpected pharmacokinetic responses. Although, the effect of any new SNPs on drugs metabolism should be confirmed by *ex vivo* and *in vivo* studies.

Our results in *10 allele (13.75%) were in agreement with Kouhi et al. (7) study about EA population. Pharmacogenetic studies can assess the correlation between genotypes and phenotypes, and consequently helps to improve prescription of recommended drugs individually, as the main application.

In this kind of studies, the purity of ethnicity is very important, however we couldn’t have many cases in each ethnicity since our institute is a referral center in the heart of country.

## Conclusion

In the current study, we identified novel polymorphism in intron 4. This SNP is known as -90 G>A in the fourth intron, of CYP2D6, which needs to be evaluated functionally by more experiments in *in vitro* enzyme assay and pharmacokinetic levels. We need to increase our knowledge in both different alleles existing in different populations and the effect of each allele or SNP on each drug metabolism in order to predict drug prescription. Therefore, a future study regarding molecular basis of the SNP is recommended to evaluate the effect of our finding on the protein, functionality. Although a neutral SNP doesn’t effect on the enzyme function, many intronic SNPs have been reported to be effective on alternative splicing or RNA stability.
